# Cerebral Modifications and Visual Pathway Reorganization in Maculopathy: A Systematic Review

**DOI:** 10.3389/fnins.2020.00755

**Published:** 2020-08-21

**Authors:** Raffaele Nuzzi, Laura Dallorto, Alessio Vitale

**Affiliations:** Eye Clinic, Department of Surgical Sciences, University of Turin, Turin, Italy

**Keywords:** maculopathy, age-related macular degeneration, juvenile macular degeneration, neurodegeneration, neuroplasticity, systematic review

## Abstract

**Background:**

Macular degeneration (MD) is one of the most frequent causes of visual deficit, resulting in alterations affecting not only the retina but also the entire visual pathway up to the brain areas. This would seem related not just to signal deprivation but also to a compensatory neuronal reorganization, having significant implications in terms of potential rehabilitation of the patient and therapeutic perspectives.

**Objective:**

This paper aimed to outline, by analyzing the existing literature, the current understanding of brain structural and functional changes detected with neuroimaging techniques in subjects affected by juvenile and age-related maculopathy.

**Methods:**

Articles using various typologies of central nervous system (CNS) imaging in at least six patients affected by juvenile or age-related maculopathy were considered. A total of 142 were initially screened. Non-pertinent articles and duplicates were rejected. Finally, 19 articles, including 649 patients, were identified.

**Results:**

In these sources, both structural and functional modifications were found in MD subjects’ CNS. Changes in visual cortex gray matter volume were observed in both age-related MD (AMD) and juvenile MD (JMD); in particular, an involvement of not only its posterior part but also the anterior one suggests further causes besides an input-deprivation mechanism only. White matter degeneration was also found, more severe in JMD than in AMD. Moreover, functional analysis revealed differences in cortical activation patterns between MD and controls, suggesting neuronal circuit reorganization. Interestingly, attention and oculomotor training allowed better visual performances and correlated to a stronger cortical activation, even of the area normally receiving inputs from lesioned macula.

**Conclusion:**

In MD, structural and functional changes in cerebral circuits and visual pathway can happen, involving both cerebral volume and activation patterns. These modifications, possibly due to neuronal plasticity (already observed and described for several brain areas), can allow patients to compensate for macular damage and gives therapeutic perspectives which could be achievable through an association between oculomotor training and biochemical stimulation of neuronal plasticity.

## Introduction

### Rationale

Macular degeneration (MD) is one of the most common causes of visual deficit (Pascolini and Mariotti, 2012). In 2010, there were 32.4 million blind people and 191 million vision-impaired people, of which 2.1 and 6.0 million, respectively, were due to macular diseases (Jonas et al., 2014), and the number is expected to increase as a consequence of the increase in life expectancy (Wong et al., 2014). Prevalence of juvenile MD (JMD) is around 0.03% (Pi et al., 2012), but it implies higher clinical and socioeconomic consequences than age-related MD (AMD) (North et al., 2014).

Although AMD (Buschini et al., 2011) and JMD (Altschwager et al., 2017) have different pathogenesis, they share central visual loss due to photoreceptor cell death (Zarbin, 2004). The damage in visual function due to MD can be very debilitating for daily activities (Scilley et al., 2002; Knudtson et al., 2005) and thus has a significant social impact. It is remarkable how some patients, in order to overcome losing central visual acuity as the result of MD, develop eccentric vision using a different point in the more peripheral retina, the so-called “preferred retinal locus” (PRL) (Vingolo et al., 2007; Verdina et al., 2013). This kind of behavior may imply a certain degree of neural reorganization (Andrade et al., 2001) and could be used and strengthened through rehabilitative exercises to improve patients’ quality of life. Moreover, new potential treatments such as pharmacological therapy, prosthesis implants, stem cell transplantation, and gene therapy could be developed on the basis of an existent potential neural plasticity or not (Gehrs et al., 2006; Bowes Rickman et al., 2013; Van Lookeren Campagne et al., 2014). The ability of the neural visual pathway to reorganize itself, even though adult cerebral plasticity remains elusive, has indeed already been highlighted in other studies (Nuzzi et al., 2017); moreover, a further evidence in favor of this possibility seems to come from studies conducted on animal models, as discussed below. It could also be assumed that some form of plastic reorganization might also happen in case of MD, through a modification of neuronal circuits. It is therefore interesting to look for evidences in the current literature regarding modifications that have occurred in the cerebral volume and cortical activation in patients suffering from these conditions: this review aims to summarize current findings in patients suffering from maculopathy in terms of structural and functional brain changes, detected with the modern techniques of neuroimaging, wondering whether such modifications may imply neuronal plasticity and whether this could have implications in current therapeutic practice.

Over the last two decades, different methods became available to investigate the central nervous system (CNS), both structurally and functionally. Functional magnetic resonance imaging (fMRI) is a neuroimaging procedure capable of detecting functional brain activities by analyzing changes in blood flow (Miki et al., 2001, 2002; Kollias, 2004). Structural brain characteristics could instead be studied using voxel-based morphometry (VBM) analysis which is computed on MRI and quantifies the gray and white matter volume (Ashburner and Friston, 2000). Moreover, surface-based morphometry (SBM), through T1-weighted morphometric analysis, can provide additional information about the brain structure (i.e., cortical thickness, curvature, and surface area) (Clarkson et al., 2011).

## Methods

In this research, we adhered to the Preferred Items for Systematic Reviews and Meta-Analyses (*PRISMA*) guidelines.

### Search Strategy

We searched MEDLINE Daily and MEDLINE (Ovid), MEDLINE In-Process and Other Non-Indexed Citations, CENTRAL (which contains the Cochrane Eyes and Vision Group Trials Register), EMBASE (Ovid), Latin American and Caribbean Literature on Health Sciences (LILACS), CINAHL (EBSCO), Trip Database, and the National Institute for Health and Care Excellence (NICE).

We used the following search string: [(“Functional magnetic resonance” OR “Functional magnetic resonance imaging” OR fMRI) OR (SBM OR “surface-based morphometry” OR “surface-based analysis”) OR (VMB OR “Voxel based morphometry” OR “Voxel based analysis”)] AND [“gray matter” OR “cerebr^∗^ OR cortex OR (visual AND (pathways OR cortex OR cortic^∗^ OR cerbr^∗^)) AND (Maculopathy OR (Macular AND disease) OR (macula^∗^ AND dystrophy) OR “macular degeneration” AMD OR JMD OR (Juvenile AND macula^∗^)].

These searches were supplemented by hand-searching the bibliographies of all the included studies. Gray literature was not considered due to excessive lack of essential information that usually affects this type of research. We did not use any data or language restrictions in the electronic searches. Articles published until March 2020 were included.

### Study Design

Study typologies included in this review were randomized controlled trials (RCTs), clinical trials, non-randomized comparative studies, cohort studies, and case series (CS).

Exclusion criteria for eligibility were case report, case series with less than six patients, and absence of outcome data.

### Participants and Interventions

We included studies on patients affected by AMD, both the exudative and the dry forms, and JMD. JMD includes a group of disease causing a loss of photoreceptor usually inherited starting early in childhood, such as Stargardt’s disease (Tanna et al., 2017), Best vitelliform retinal dystrophy (Blodi and Stone, 1990), cone-rod dystrophy, and central areolar choroidal dystrophy (Xiao et al., 2010; Bondarenko et al., 2014; Molday, 2015; Palejwala et al., 2016; Hohman, 2017). Patient characteristics are reported in Table 1. The following techniques were used for investigating brain changes:

**TABLE 1:** Demographic and clinical characteristics.

**Author, year**	***N* patients**			**Mean age (range)**			**Sex (M/F%)**			**Clinical characteristic**	
	**AMD**	**JMD**	**H**	**AMD**	**JMD**	**H**	**AMD**	**JMD**	**H**	**AMD**	**JMD**
Baker et al., 2008	0	7	7	NS	NS	NS	NA	57/43	NS	NA	Bilateral large central bilateral scotoma (5 with no foveal function, 2 with foveal function)
Baseler et al., 2009*	8	8	12 (O 7 Y 5)	76 (70–90)	30 (19–49)	O (61–77) Y (18–37)	50/50	38/62	NS	Bilateral 1-year central scotoma with stable PRL	Stargardt’s disease with bilateral 1-year central scotoma with stable PRL
Boucard et al., 2009	9	0	12	73 (61–85)	NA	66 (60–82)	78/22	NA	75/25	Bilateral scotoma >10° for minimum 3 years	NA
Haak et al., 2016*	0	8	12	NA	30 (19–49)	(18–41)	NA	38/62	NS	NA	Stargardt’s disease with bilateral 1-year central scotoma with stable PRL
Hernowo et al., 2014*	24	34	55 (O 22 Y 33)	75.2 (52–91)	40.2 (12–66)	O 68 (61–83) Y 37 (13–60)	58/42	62/38	46/54	Binocular VF defect (mean scotoma 14°)	Binocular VF defect (mean scotoma 20°)
Lešták et al., 2013	10	0	9	74.7 (58–85)	NA	54.1 (45–65)	10/90	NA	78/22	Wet form of AMD with various degrees of bilateral impairment	NA
Little et al., 2008	6	0	12 (O 6 Y 6)	(55–83)	NA	O 25 (22–31) Y 73 (54–78)	50/50	NA	O 67/33 Y 50/50	Bilateral GA with eccentric PRL	NA
Liu et al., 2010	4	4	2	(70–90)	(30–50)	(70–90)	NA	NA	NA	More than 10 years AMD with stability of PRL for at least 5 years	More than 10 years Stargardt’s disease
Olivo et al., 2015	0	18	23	NA	30.6 (15–54)	30.8 (18–60)	NA	67/33	65/35	NA	Clinical and molecular diagnosis of Stargardt’s disease
Plank et al., 2011*	0	26	26	NA	42 (12–66)	43 (13–70)	NA	69/31	50/50	NA	Binocular central scotoma >10° and VA <0.2 decimal
Plank et al., 2013*	0	22	22	NA	41.5 (12–65)	42.4 (13–70)	NA	64/36	50/50	NA	Hereditary retinal dystrophies with binocular absolute central scotoma >10° and VA <0.2 decimal
Plank et al., 2014	8	5	12	66.8 (58–79)	59 (47–69)	62.1 (47–78)	50/50	80/20	33/67	Minimal duration of disease: 5 years, scotoma size: 10–25°	3 patients with cone-rod dystrophy and 2 patients with Stargardt’s disease. Scotoma size: 10–15°
Plank et al., 2017*	0	19	19	NA	41.11 (13–65)	41.74 (13–70)	NA	58/42	53/47	NA	Hereditary retinal dystrophies with binocular absolute central scotoma >10° and VA <0.2 decimal
Prins et al., 2016*	24	34	55 (O 22 Y 33)	75.2 (52–91)	40.2 (12–66)	O 68 (61–83) Y 37 (13–60)	58/42	62/38	46/54	Binocular VF defect (mean scotoma 14°)	Binocular VF defect (mean scotoma 20°)
Rosengarth et al., 2013	9	0	7	72 (55–84)	NA	69 (51–83)	45/55	NA	29/71	Scotoma size >10°	NA
Schumacher et al., 2008	5	1	7	73.5 (63–82)	NA	75.2 (63–82)	67/33	NA	83/17	5 AMD + 1 JMD	NA
Szlyk and Little, 2009	6	0	12 (O 6 Y 6)	72 (55–83)	NA	O 73 (54–78) Y 25 (22–31)	50/50	NA	Y 50/50 O 67/33	Bilateral GA with eccentric PRL	NA
Whitson et al., 2015	7	0	32 (O 16 Y 16)	79.9 (69–91)	NA	O 68.3 (65–74) Y 23.5 (18–35)	14/86	NA	50/50	Long-standing bilateral AMD	–
Masuda et al., 2008	0	4	3	NA	37 (22–57)	(29–33)	NA	3/1	3/6	NA	2 cases of Stargardt’s Disease and 2 cases of cone-rod dystrophy, with central-bilateral scotoma

*Baseler et al. (2011), Hernowo et al. (2014), Haak et al. (2016), and Prins et al. (2016) share the same patients, Plank et al. (2011, 2013, 2017) shared some of the patients median value of age. Y, young; O, old; NS, not specified; NA, not applicable; PRL, preferred retinal locus; VF, visual field; AMD, age-related macular dystrophy.

– fMRI
– VBM
– SBM

### Data Sources, Study Sections, and Data Extraction

The data were collected independently by two reviewers.

The research resulted in 142 articles (37 from *PubMed*; 39 from *EBSCO*; 52 from *Embase*; 11 from *Trip Database*; and 3 from free search). Thirty duplicate results were removed using the *EPPI* reviewer (by EPPI-Center, Social Science Research Unit, the Institute of Education, the University of London, United Kingdom). Screening of titles and abstracts was then carried out, and no pertinent articles were rejected: after this selection, 22 articles were assessed for eligibility. Three articles were later rejected due to exclusion criteria, with a final number of 19 articles.

Full texts of these articles were evaluated independently.

**FIGURE 1:**
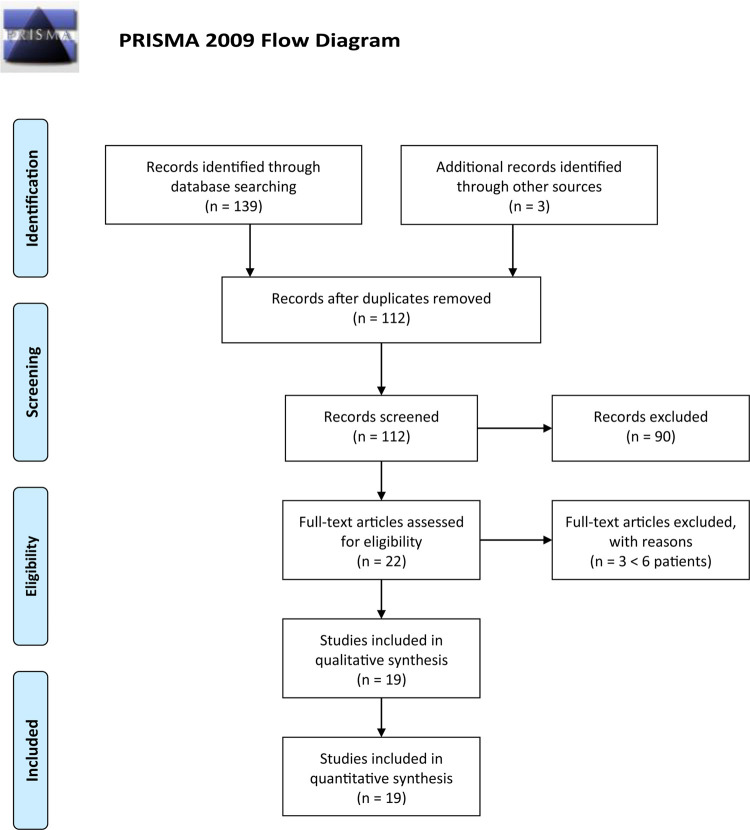
PRISMA flow diagram.

The entire process was carried out according to the PRISMA flow diagram: details on screening process are given in Figure 1.

### Data Analysis

Analyzed parameters are listed below:

– VBM: gray and white matter volume.
– SBM: morphologic characteristic of the brain cortex such as thickness, curvature, and surface area.
– fMRI: blood oxygenation level-dependent (BOLD) evaluation. Changes in hemoglobin oxygenation in focal blood flow are used to detect neuronal activity fluctuation.

## Results

### Study Characteristics

Five out of 19 (26%) studies examined brain changes in terms of structural parameters (one study used SBM and four used VBM). The remaining 14 studies (74%) used fMRI to evaluate cortical response changes following MD.

All included studies were case series with a sample ranging from 6 to 58 patients; patient characteristics are reported in Table 1. Subjects showed high heterogeneity in mean age, disease duration, and disease stage.

Six studies (31.5%) included only AMD patients, seven studies included only JMD patients, and the remaining six studies included both AMD and JMD patients.

### Outcomes of Structural Analysis (Table 2)

Four studies (Boucard et al., 2009; Plank et al., 2011; Hernowo et al., 2014; Olivo et al., 2015) investigated brain changes using VBM in 33 patients with AMD and 78 with JMD: two of them (Hernowo et al., 2014; Olivo et al., 2015) reported results on both gray and white matter, while the other two studies (Boucard et al., 2009; Plank et al., 2011) showed results on gray matter only.

**TABLE 2:** Outcomes of structural analysis.

**Author, year**	**Methods**	**Parameter analyzed**	**Area analyzed**	**Outcomes**	**Principal results**		**Secondary results**
					**AMD**	**JMD**	
Boucard et al., 2009	VBM	Gray matter density in AMD, POAG, controls	(1) Whole brain (2) VOI (21 mm diameter) at the occipital pole: posteriorly and anteriorly in the calcarine cortex, corresponding to foveal and peripheral visual field projection zone respectively	Differences compared to age-matched groups and to glaucoma group	(1) Gray matter density of occipital pole, especially around calcarine fissure, is reduced (primarily in the left hemisphere). (2) Gray matter density is more reduced in the posterior than in the anterior region, corresponding to the foveal projecton zone.	NA	POAG patients showed a more anterior reduction in the occipital cortex, corresponding to the peripheral visual field projection zone
Plank et al., 2011	VBM	Gray matter volume and density in patients with central scotoma due to hereditary retinal dystrophies	(1) Regional differences (2) 4 VOI along the calcarine sulcus. (4 VOIs of 5 mm radius from posterior to anterior)	Differences compared to age-matched groups	NA	(1) Significant reduction of gray matter volume around the calcarine sulcus of both hemispheres. (2) In both hemispheres VOI was more reduced in the posterior part of calcarine sulcus than anterior part. (3) Density analyses results was comparable to those founded in volume analyses.	(1) Scotoma size negatively correlated with GM volume in VOIs 2 and 3. (2) Reading speed positively correlated to GM volume of the more anterior VOI in the left hemisphere. (3) Fixation stability positively correlated with GM volume of a cluster in the right superior frontal gyrus.
Olivo et al., 2015	VBM	Gray and white matter volume in pz. Suffering from in Stargardt’s disease	Regional differences	Differences compared to age-matched groups	NA	(1) Gray matter loss bilaterally in the occipital cortices (with a greater extension on the right one). (2) Smaller cluster of GM loss in the adjacent parietal lobe on the right one (within the precuneus).	Gray matter volume correlated directly with mean visual sensitivity in the right middle frontal and left calcarine gyri, and inversely with retinal thickness in the left supramarginal gyrus.
Hernowo et al., 2014*	VBM	Gray and white matter volume in AMD and JMD	(1) Whole brain (2) 5 ROI: PGCL (optic nerve, chiasm and optic tract), LGBs, GCRs, OCP, CCR (intracalcarine and supracalcarine cortices)	Differences compared to age-matched groups	(1) Reductions of gray and white matter located in the visual cortex and optic radiations; reduction of white matter in the frontal lobe. (2) Volume reduction throughout all visual pathway: GCR, OCP, CCR.	(1) Reductions of gray and white matter located in the visual cortex and optic radiations. (2) Volume reduction throughout all visual pathway: LGB, GCR, OCP.	(1) Reductions, specially of white matter, is more pronounced in the AMD group. (2) Volume of LGB, OCP, CCR reduces with age (3) No correlation between ROI volume and disease duration.
Prins et al., 2016*	SBM	Gray matter thickness, mean curvature, surface area and volume in AMD and JMD	(1) Whole brain (cortical thicknesses and mean curvature) (2) ROI: V1 and V2 areas, both divided in anterior and posterior	Differences compared to age-matched groups	(1) Whole brain: No differences. (2) ROI: thinner cortex in V2 anterior and V2 posterior. No differences in mean curvature, surface area size and gray matter volume.	(1) Whole brain: No differences. (2) ROI: thinner cortex in V1 posterior and V2 posterior, smaller surface area in V1 anterior, V1 posterior and V2 posterior and lower gray matter volume in V1 posterior, V2 anterior and V2 posterior. No differences in mean curvature.	No correlation between structural parameters in the ROIs or whole image and disease duration

VOI, volume of interest; POAG, primary open angle glaucoma; ROI, region-of-interest; PGCL, pregeniculate structures; GCR, geniculocalcarine radiations; LGB, lateral geniculate body; OCP, the occipital pole; CCR, calcarine region; GM, gray matter; MS, mean sensitivity; CRT, central retinal thickness. *Hernowo et al. (2014) and Prins et al. (2016) share the same patients.

Boucard et al. (2009) compared gray matter density in 9 AMD, 8 primary open angle glaucoma (POAG), and 12 healthy subjects. In particular, VBM was used to assess local changes in gray matter density in the whole brain and in specific volumes of interest (VOIs): 21-mm-diameter spheres in each hemisphere posterior and anterior to the calcarine cortex, corresponding to the foveal and the peripheral visual field projection zones, respectively. In the AMD subjects, gray matter density was reduced mainly in the posterior part; POAG patients showed instead a more anterior reduction in the occipital cortex. These data demonstrated a retinotopically gray matter damage caused by late-onset acquired visual field defect.

Plank et al. (2011) used VBM to analyze structural brain changes in 26 patients with central scotoma due to hereditary retinal dystrophies. Patients with JMD showed a significant reduction of gray matter volume in the posterior part of calcarine sulcus in both hemispheres. Gray matter density analysis produced results comparable to those about gray matter volume. Correlation analysis showed a negative correlation between scotoma size and gray matter volume in central VOI along the calcarine sulcus. Reading speed positively correlated to gray matter volume in the more anterior part of the calcarine sulcus in the left hemisphere, while eccentric fixation stability positively correlated with gray matter volume of a cluster in the right superior frontal gyrus.

Similar results came from Olivo et al. (2015) who used VBM to analyze gray and white matter volume in Stargardt’s disease. A gray matter loss was demonstrated bilaterally in the occipital cortex and in the adjacent parietal lobe. Moreover, gray matter volume correlated inversely with retinal thickness in the left supramarginal gyrus: since, among other tasks, this part controls oculomotor mechanisms, it is possible to assume a compensatory role for this occurrence, in terms of adaptive oculomotor learning.

Hernowo et al. (2014) investigated changes in both white and gray matter of 24 AMD and 34 JMD patients: whole-brain analysis showed a reduction of gray and white matter located in the visual cortex and optic radiations. Volumetric reduction was demonstrated throughout all the retrobulbar afferent structures, with some differences: JMD patients showed a reduction all along the visual pathway; on the other hand, in AMD patients, only the lateral geniculate bodies and the optic radiations were affected. Also, volumetric reduction in JMD was greater than that in AMD, maybe because of the larger functional damage that JMD subjects had. Moreover, in the AMD group, volumetric reduction of white matter was also demonstrated in the frontal lobe, but it might be related to the different ages of AMD and JMD patients.

Only one study (Prins et al., 2016) analyzed cortical changes using SBM. This study was conducted on the same patients analyzed with VBM by Hernowo et al. (2014): volumetric changes, detected in MD patient’s visual cortex using VBM, were further characterized by a more accurate representation of structural differences. The surface area in the anterior region of the visual cortex was found to be reduced. Another result was the more widespread structural damage in terms of cortical thinning and the greater reduction of surface area and volume in JMD than in AMD patients; this result could be, however, biased by the larger visual field defect and poorer visual acuity in JMD patients, resulting in greater visual deprivation. No structural changes outside the visual cortex were found, nor was there a significant correlation between disease duration and structural parameters.

### Outcomes of Functional MRI (Table 3)

Fourteen studies used fMRI in order to investigate functional changes in brains of patients suffering from AMD or JMD.

**TABLE 3:** Outcomes of functional analysis.

**Author, year**	**Methods**	**Parameter analyzed**	**Outcomes**	**Principal results**		**Secondary results**
				**AMD**	**JMD**	
Little et al., 2008	fMRI	ROI activation patterns during eye movements in AMD patients and controls. (ROI: FEFs, SMA/SEFs, IPS, IPS, V1, V2/V3, MT/V5, PFC)	To examine the cortical networks that underlie saccadic and pursuit eye movements in patients affected by AMD who use PRLs and to compared to old and young groups.	Decreased activation in visual cortex. Increased activation of FEFs, PFC, IPS, and SMA/SEFs	NA	–
Szlyk and Little, 2009	fMRI	ROI activation during 3-letters and 6-letters word recognition in AMD patients using PRLs vs controls (ROIs: left and right frontal eye fields; supplementary motor areas, superior parietal lobule, left and right inferior parietal lobule, primary visual cortices; secondary and tertiary visual cortex; fusiform gyrus; prefrontal cortex)	Existing activation differences compared to control	Patients showed increased brain activation in frontal eye fields, superior and inferior parietal lobules, and regions within the prefrontal cortex. Peak activation within these prefrontal regions was correlated with increased accuracy and decreased reaction times for the 3-letter task within the group of patients. Correlations between peak activity and behavioral performance were also found in both the right and left superior parietal lobules for the 3-letter task	NA	–
Plank et al., 2013	fMRI + structural MRI	Visual cortex activation (V1–V3 areas) in JMD patients vs controls during retinotopic mapping and visual tasks	Existing activation differences compared to control	NA	Patients with an established PRL had higher activation in early visual cortex during the visual search task, especially when the target stimuli fell in the vicinity of the PRL. Patients with stable eccentric fixation at the PRL exhibited greater performance levels and more brain activation compared with those with unstable eccentric fixation	–
				**AMD**	**JMD**	
Plank et al., 2014	fMRI	Visual cortex activation (V1–V3 areas) in AMD and JMD patients, compared with controls, after training	Cortical activation changes after visual training at eccentric PRL to perform a challenging TDT	Both patients and control subjects exhibited a typical learning effect on the TDT. Training on the TDT enhances eccentric vision in patients with central vision loss, allowing better performances in the task. This enhancement is accompanied by an increased response in in the PRL projection zone of the visual cortex.		–
Plank et al., 2017	fMRI	Activation of visual cortex and other ROIs during stimulation of PRL and oppPRL with flickering check board or object pictures, in JMD. (ROIs: areas 17-18 Brodmann; lateral occipital complex, fusiform cortex, and inferior temporal grus)	Differences in cortical activation by stimulating PRL and oppPRL.	NA	(1) PRL stimulation, especially in patients with highly stable eccentric fixation, caused activation of early visual cortex and higher visual areas. (2) PRL stimulation caused higher activation than oppPRL (3) PRL stimulation produced coactivation of the central representation area in early visual cortex in patients, but not in controls.	Stimulation with everyday objects led to larger responses than flickering checkerboards
Rosengarth et al., 2013	fMRI	Cortical activation and Gray and White matter density changes after fixation training in AMD patients and controls	Existing differences before/after training, and in comparison to control	(1) Positive correlation between achieved fixation stability and cortical response in the PRL-projection zones. (2) Increments in Gray and White matter density in the cerebellum after training	NA	–
Lešták et al., 2013	fMRI	Visual cortex activation by retinal stimulation	Existing differences in cortical responses between AMD patients and controls	Lower fMRI activity of the visual cortex in wet form of AMD compared with the control group.	–	Dependence of fMRI activity on visual acuity was not statistically significant
				**AMD**	**JMD**	
Liu et al., 2010	fMRI	Visual cortex activation by retinal stimulation (Six ROIs along the calcarine sulcus, from posterior pole of the occipital cortex to anterior part of the calcarine)	Existing differences in cortical activation generated by stimuli at PRL or non-PRL locations in AMD, JMD and controls.	(1) Small stimulus at the PRL generated more extensive cortical activation than at a non-PRL location, but no activation in the fovea cortical projection. (2) Both passive and active viewing of full-field stimuli left a silent zone at the posterior pole of the occipital cortex, even if smaller in case of active viewing during a visual task, especially in subject suffering from JMD Since activity in cortical areas corresponding to central scotoma projection is considered evidence for functional reorganization, these results suggested a certain degree of functional reorganization in early visual cortex in both JMD and AMD, even if not complete, and apparently more prominent in JMD.		–
Baseler et al., 2011	fMRI + structural MRI	Cortical response of calcarine sulcus (peripheral retina), occipital pole (fovea) and non-visual cortex after passive visual stimulation	To compare responses presented by AMD patients, JMD patients and controls. All patients using PRLs.	(1) No difference in response of lesion projection zone between patients and controls (simulating scotoma) (2) ‘Ectopic’ receptive fields areas were detected in lesion projection zone equally in both patients and controls Questioning if responses in V1 in patients with macular degeneration has to be explained by occurred remapping or as an effect given by neuronal feedback and lateral connections		Findings were not dependent on the age at which the individuals acquired retinal lesions
Masuda et al., 2008	fMRI	Visual cortex activation (V1 area, particularri LPZ)	To determine cortical response to different visual stimuli (checkerboards, drifting contrast patterns, scrambled, intact faces) directed toward PRL (in JMD) or a retinal location that corresponded to PRL (in controls)	NA	Significant responses were observed in the LPZ only when performing stimulus-related tasks. No significant cortical activation was recorded during passive viewing or visual tasks unrelated to stimulus In controls peripherap retinal stimulation did not produce response in foveal projection zone	–
				**AMD**	**JMD**	
Baker et al., 2008	fMRI	Measure the magnitude of response in cortical regions corresponding to the representation of the fovea in occipital pole.	To compare the response to stimuli presented at the fovea and PRL in JMD and AMD (divided in foveal damage and fovea residual function) and control groups.	(1) MD with foveal damage: stimuli presented at the PRL elicited strong responses in the occipital pole while stimuli presented at the fovea elicited little or no response. (2) MD with fovea residual function: stimulation of the fovea produced weak activation of the occipital pole. Stimulation of PRL produced no activation. (3) Control participants: no activation of investigated areas for stimuli presented to peripheral retina (corresponding to the matched MD participant’s PRL).		–
Whitson et al., 2015	fMRI	Activation of ROI involved in networks that support executive function, language and/or vision during a phonemic fluency test in AMD patients (ROIs: Left opercular portion of inferior frontal gyrus, left superior temporal gyrus, inferior parietal lobe, right superior parietal lobe, right supramarginal gyrus, right supplementary motor area, right precentral gyrus)	To examine the relationship between fMRI measures of rsFC (*Resting-state functional connectivity)*, across several preselected functional networks, and behavioral assessment of phonemic fluency (obtained prior to the scanning session) in individuals with AMD	AMD subjects phonemic fluency resulted inversely related to the existing connectivity among these areas: patients with stronger connections exhibited worse fluency. In controls there was no correlation between connectivity and fluency.	NA	–
Schumacher et al., 2008	fMRI	Activation of visual cortex (ROI: alcarine sulcus)	To measure brain activity in calcarine sulcus while visually stimulating PRL, non-PRL in AMD/JMD patients and corresponding retinal areas in controls	MD patients showed more brain activity in posterior calcarine sulcus (i.e., the foveal confluence) in response to visual stimulation of their PRL than in response to stimulation of other visual field sections (e.g., non-PRL), or corresponding retinal areas in age-matched controls		–
Haak et al., 2016	fMRI + connective modeling	BOLD responses and connective field modeling which estimates a voxel’s population receptive field (pRF) toward estimating a voxel’s connective field.	To determine whether the functional connectivity between the input-deprived portions of V1 and V2/3 is still retinotopically organized.	NA	Functional connectivity between the input-deprived portions of visual areas V1 and extrastriate cortex is still largely retinotopically organized in MD, although on average less than in controls.	–

*FEFs, frontal eye fields; SMA/SEFs, supplementary eye fields; IPS, intraparietal sulci; V1, primary visual area; V2/V3, secondary and tertiary visual area; PRL, Preferred Retinal Locus; oppPRL, retinal area comparable to PRL but in the opposite hemifield; TDT, texture discrimination task; MT/V5, visual motion regions; PFC, prefrontal cortex; LPZ, Lesion Projection Zone.*

Little et al. (2008), analyzed several region-of-interest (ROI) activation patterns during standardized eye movements (saccade and smooth pursuit) in patients suffering from AMD. ROIs included frontal and supplementary eye fields (FEFs and SMA/SEFs), prefrontal cortex (PFC), intraparietal sulci (IPS), and visual cortex areas (V1/V2/V3 and MT/V5). Results showed a marked reduction of activity in the visual cortex but an increased activity in FEFs, PFC, IPS, and SMA/SEFs, if compared with those of control subjects. Patients with AMD can try to overcome the impaired visual function using paracentral PRLs: the incongruence between gaze direction and visual input elaboration, normally closely associated, results mostly in difficult performance of oculomotor tasks, with a high impact on everyday activities such as reading; the attempt to overcome this incongruity, including through a learning path and involving other areas of visual control, can justify the detected pattern of activation.

Szlyk and Little (2009) studied cortical activation in patients suffering from AMD who learned to use a PRL to overcome central vision loss, during a visual task of three-letter and six-letter word recognition: if compared with controls, patients showed an increased brain activation in many areas such as the frontal eye fields, parietal lobules, and prefrontal cortex. As a possible explanation of these findings, it was assumed that not only the decreased sensory input but, rather, also the continuous research for the visual target required in a situation of central visual field deficit was causing an increased involvement of cognitive resources, among which attention has a great importance, justifying the higher activation of these superior cortical areas in these patients.

Plank et al. (2013) studied visual cortex activation (V1, V2, and V3) in JMD patients using fMRI, during retinotopic mapping and visual tasks. Patients with an established PRL exhibited significantly higher activation in the primary visual cortex during the visual search task, especially when the target stimuli fell near the PRL. Compared with those with less stable fixation, patients with firm eccentric fixation at the PRL exhibited greater performance levels and higher brain activation. Plank et al. (2014) an fMRI study in 8 AMD, 5 JMD, and 12 controls from the same group showed that visual exercise [in this case, a texture discrimination task (TDT)] focused on PRL can enhance eccentric vision in patients with central vision loss. Both patients and control subjects exhibited a learning effect on the TDT, enhancing eccentric vision ability and so allowing better performances. This enhancement was also accompanied by an increased response in the PRL projection zone of the visual cortex.

Plank et al. (2017) used fMRI on JMD patients and controls to examine whether a visual stimulation of PRL would have caused a greater activation of the visual cortex in comparison with stimulating an equivalent peripheral area in the opposite hemifield (oppPRL). Retinal loci were stimulated using a flickering checkerboard and real-object pictures during fMRI measurement. Stimulation of PRL produced coactivation of the central representation area in the early visual cortex in patients, but not in controls. Moreover, especially in patients with highly stable eccentric fixation, it caused a stronger activation of the early visual cortex and also a response by higher visual areas beyond the retinotopic cortex. In addition, cortical response given by PRL was more intense than the one triggered by stimulating the oppPRL. Lastly, stimulation with everyday objects led to overall larger responses than those evoked by flickering checkerboards.

Rosengarth et al. (2013) observed changes in gray and white matter density due to fixation training in nine cases of AMD and seven controls. They also found a positive correlation between the increasing fixation stability and cortical response in the PRL projection zone. Structural changes were also found in the cerebellum of patients following oculomotor training: increments in gray and white matter density in the cerebellum might be the consequence of the learning effect achieved by performing visual tasks using eccentric viewing. Thus, this study showed how patients with AMD can actually benefit from specific oculomotor training procedures and how these improvements correspond to functional and structural changes in the CNS.

Lešták et al. (2013) found that patients with a wet form of AMD showed, during retinal light stimulation, a lower fMRI activity of the visual cortex compared with the control group of healthy subjects. However, the dependence of the neurological activity on visual acuity was not statistically significant.

Liu et al. (2010) studied cortical activation in four cases of AMD, four cases of JMD, and two controls using fMRI. Six ROIs were chosen along the calcarine sulcus, extending from the posterior pole of the occipital cortex to the anterior part of the calcarine sulcus in each hemisphere. The results of this study demonstrated that a stimulation of PRL generated more extensive cortical activation than non-PRL, but no activation in the foveal cortical projection was detected. Both passive and active viewing of full-field stimuli left a silent zone at the posterior pole of the occipital cortex, even if smaller in case of active viewing during a visual task, especially in subjects suffering from JMD. Since activity in cortical areas corresponding to central scotoma projection is considered an evidence of functional reorganization, these results suggested a certain degree of remodeling in the early visual cortex in both JMD and AMD, even if not complete and apparently more prominent in JMD.

Baseler et al. (2011), studied 8 AMD cases, 8 JMD cases, and 12 control subjects with fMRI and structural MRI, recording cortical activity in early visual areas (V1–V3) of occipital pole during visual stimulation. All affected subjects had developed a stable PRL that allowed good eccentric fixation. Stimulation was based on passive viewing, without any active visual tasks requested. Their results ran contrary to the belief that the visual cortex could reorganize itself after retinal damage and input deprivation to respond to peripheral retinal stimulation: the cortical area corresponding to the lesion projection zone (LPZ) gave activity signals in the patient group in a different way than in the control group in whom a central scotoma was simulated. Furthermore, these findings were not dependent on the age at which the individuals acquired retinal lesions.

Moreover, areas with “ectopic” receptive fields were detected throughout the LPZ, equally representing the same regions of visual field in both patients and controls and questioning if the existence of ectopic receptive fields can continue to be considered as an evidence of remapping that occurred or, instead, a simple effect given by neuronal feedback and lateral connections.

It is worth noting that this study used a passive visual stimulation, and not a visual task, that has already been proven in others to be an important factor for the response of the LPZ in the early visual cortex (Masuda et al., 2008; Plank et al., 2013, 2014). Baseler hypothesized however, that these findings could be justified by the existence of feedback from the extrastriate cortex and/or the absence of feedforward signals from the visual cortex, rather than by a remapping that has occurred. He concluded there was no need to invoke remapping as an explanation of responses in V1 of individuals with MD.

Masuda et al. (2008) studied LPZ responses to different visual stimuli (checkerboards, drifting contrast patterns, scrambled, and intact faces) in four cases of Stargardt’s disease and cone-rod dystrophy with central–bilateral scotoma and three controls, using fMRI. JMD subjects viewed a fixation point at the center of the stimulus using their PRL, while in controls, the fixation point fell in a retinal locus corresponding to patients’ PRL. LPZ activation was indeed observed, but only as a consequence of stimulus-related tasks: any activity was recorded during passive viewing or visual tasks unrelated to stimulus. In control subjects, in which visual stimulus was presented only within the peripheral visual field, there was no response in the foveal projection zone in any condition. The study concluded that LPZ responses are not simply stimulus driven but rather are resulting from stimulus-related tasks: these findings may depend on new cortical pathways carrying task-dependent signals (neural reorganization) or the unmasking of preexisting task-dependent cortical signals that ordinarily are suppressed by the retinal visual input (no reorganization).

Baker et al. (2008) conducted a study in which seven subjects affected by JMD or AMD and seven healthy controls viewed blocks of images, recording activation of the occipital cortex using fMRI. In two of the affected subjects, MD preserved foveal function, while the other ones used a PRL. Images were presented at either a foveal or a peripheral retinal location (PRL or a matched location in controls). After PRL stimulation, patients with foveal damage elicited strong responses of cortical regions corresponding to the foveal representation, while stimuli presented at the fovea evoked little or no response. On the other hand, subjects with still foveal residual function produced a response after its stimulation, even if weak, while stimulation of a corresponding PRL produced no activation of these areas. In controls, stimuli presented to the corresponding PRL retinal point did not produce any response in the analyzed areas; on the contrary, stimuli presented at the fovea produced a strong response. The conclusion was that large-scale reorganization of visual processing occurs only in the complete absence of functional foveal vision.

Whitson et al. (2015) examined the relationship between vision impairment caused by AMD and poor performance on phonemic fluency tests, using fMRI in seven subjects with long-standing bilateral AMD. In particular, activation of several ROIs was investigated: left opercular portion of inferior frontal gyrus (which includes Broca’s area), left superior temporal gyrus (which includes part of Wernicke’s area), inferior parietal lobe, right superior parietal lobe, right supramarginal gyrus, right supplementary motor area, and right precentral gyrus. All these areas are involved in networks that support executive function, language, and vision during a phonemic fluency test. In AMD subjects, phonemic fluency was inversely related to the existing connectivity among these areas, since patients with stronger connections exhibited worse fluency; meanwhile, in controls, there was no correlation between connectivity and fluency. So compared with controls, patients showed stronger connectivity within these areas, but these findings were not an unequivocal explanation: it was not clear if they simply reflected the increased cognitive effort required to support this kind of task in the context of non-specific age-related brain changes or, rather, some compensatory changes to support cortical elaboration in a setting of reduced vision.

Schumacher et al. (2008) evaluated calcarine sulcus activity during visual stimulation in AMD and JMD patients compared with controls using fMRI. Cortical response was recorded during stimulation of both PRL and non-PRL retinal areas in patients, and peripheral retinal loci in controls, by using a checkerboard pattern. Cortical activity of the posterior calcarine sulcus in response to visual stimulation of PRL was higher than that of non-PRL in patients and than that of PRL-corresponding regions in a group of age-matched controls, possibly implying that a large-scale cortical reorganization of visual processing occurred in response to MD.

Haak et al. (2016) studied 8 patients with Stargardt’s disease and 12 healthy controls, using fMRI and connective field modeling, to establish whether connectivity between input-deprived portions of the primary visual cortex (V1) and early extrastriate areas (V2/V3) was still retinotopically organized. The study showed that functional connectivity between V1 and V2/V3 was still largely retinotopically organized, although less than that in controls, concluding that despite the prolonged lack of visual input due to MD, corticocortical connections of the primary visual cortex remained largely intact.

## Discussion

Hereditary and age-related macular dystrophies, diseases with great epidemiological and social weight, lead to a loss of photoreceptors in the macular area (Holz et al., 2004; Takahashi et al., 2016; Chirco et al., 2017), resulting in central visual field defect (Scullica and Falsini, 2001; Gehrs et al., 2006; Cassels et al., 2017) and implying an inadequate input from this part of the retina to the visual cortex. It has been largely debated whether this could lead to a change and to a reorganization of the retinotopic maps in the visual cortex, thanks to neuronal plasticity (Kitajima et al., 1997; Dreher et al., 2001; Sunness et al., 2004; Baker et al., 2005; Baseler et al., 2009; Dilks et al., 2009, 2014; Masuda et al., 2010; Chung, 2013; Barton and Brewer, 2015). Actually, the ability to adapt to lesions by a mechanism of neuronal plasticity has already been demonstrated in the CNS of both young subjects and, although to a lesser degree, adult subjects (Rosa et al., 2013). So there are reasons to question whether such an eventuality can also occur in cerebral visual areas. A clue in favor of such a thesis could come from studies conducted on animal models: experiments concerning reorganization of the primary visual cortex following retinal lesions has been performed by several authors.

Kaas et al. (1990) analyzed cortical modifications in adult cats after iatrogenic lesion of the central retinal area of one eye and enucleation of the other eye, recording single-neuron responses from the primary visual cortex: it was detected that neurons originally receiving inputs from the central retina became responsive to stimuli presented to intact peripheral retinal locations surrounding the destroyed area. This result can suggest a reorganization of the visual cortex: neurons in the deprived zone of the cortex acquired new receptive fields representing inputs originating from retinal locations around the margins of the lesion.

Similarly, Heinen and Skavenski (1991) described that in adult monkeys, after bilateral macular lesions, cortical neurons in foveal representation became responsive to visual stimuli presented to intact peripheral retina 2.5 months after lesions.

Again, Botelho et al. (2014) observed how monocular lesions in monkeys caused a significant reorganization of the topographic map in the visual area, both inside and outside the cortical LPZ, starting immediately after the retinal lesion. The short time frame observed in this case between macular lesion and cortical reorganization allowed us to assume the preexistence of intracortical connections in V1 unmasked by cortical deafferentation.

Lastly, mouse visual cortex structural modifications, represented by a rapid and lasting reduction in the number of cell spines of inhibitory neurons after removal of visual input, were described by Keck et al. (2011).

### Analysis of Structural Results

All reviewed studies found cerebral structural changes in terms of reduced volume following MD, suggesting how a central scotoma, even if acquired later in life, can lead to transsynaptic degeneration. These modifications involved both gray and white matter, eventually all along the entire visual pathway from the optic nerve to the cortex. These results are consistent with the current knowledge that gives to the neuronal afferents not only a task of transmission of information but also a trophic effect toward target cells. A structural consequence is therefore undoubted, but with the volumetric analysis alone, it is difficult to understand if, along with a variation due to the ceased trophic effect by afferent innervation, there is also a variation related to a reshaping/reorganization. Some structural results are worthy of attention:

– Regarding visual cortex volume, a reduction in both anterior and posterior thickness was recorded in AMD as well as JMD patients. So, as central vision is represented in the posterior region of the visual cortex, not only could cortical damage be related to a degeneration due to retinotopic-specific deprivation, but further explanations of the anterior modifications could also be given by an effect of spontaneous oscillatory spike bursts that retinal ganglion cells were demonstrated to produce after retinal degenerations, as well as by a changed function that the more peripheral retina acquires in MD patients due to the selection of a new PRL to obtain an eccentric fixation (Prins et al., 2016).
– Moreover, in the comparison of the results between AMD and JMD groups, some differences emerged (Hernowo et al., 2014; Prins et al., 2016): in JMD, a more extensive damage was identified in terms of both involved structure amount (the whole optical pathway, from the optic nerve to the cortex, while only from lateral genicolate bodies in AMD) and volumetric reduction. In the two reference studies, however, this outcome was considered, rather than as a marker of reshaping, as the effect of JMD patients having more severe retinal alterations and greater visual deficits if compared to AMD patients.
– Potential structural indicators of remodeling that occurred come from Plank et al. (2011) and Boucard et al. (2009): an increased volume at the level of supramarginal gyrus, involved in oculomotor coordination, and a positive correlation between gray matter in right superior and middle frontal gyri and fixation stability were findings that could be the consequence of oculomotor learning in patients with central scotoma, forced to use their own peripheral visual field to obtain an eccentric fixation.

### Analysis of Functional Results

Studies using functional imaging to analyze cortical activity in MD patients produced variable results, generating some controversy:

– All studies have shown changes in cortical activity; in particular, the visual cortex activation pattern was different and less intense if compared to healthy controls (Lešták et al., 2013). It should be noted, however, that in addition to the expected changes in the occipital lobe, there was also an increase in activity of frontal brain areas (frontal eye fields, supplementary eye fields, prefrontal cortex, and intraparietal sulci), classically associated with attention (Little et al., 2008; Szlyk and Little, 2009): this suggests that an adaptation of the brain occurred, although not clearly indicative of remodeling. It can be assumed that, in patients trying to overcome the impaired visual function using paracentral PRL, the incongruence between gaze direction and visual input elaboration, normally closely associated, results in an increased activation of attentional networks as a compensatory behavior and, thus, that integrity of these cerebral areas is necessary for the use of PRL as a new main point of visual processing outside the diseased fovea.
– Oculomotor training, mainly aimed at the use of a PRL to obtain stability in eccentric fixation, has a positive effect on the performances obtained in the visual task (Plank et al., 2014). These effects are accompanied both by structural changes in the cerebellum (Rosengarth et al., 2013) and by interesting functional changes, where PRL stimulation generates a response of the LPZ, and greater fixation stability corresponds to a greater consequent activation (Plank et al., 2017).
– In multiple occasions, PRL stimulation was investigated: in patients using PRL for visual elaboration, its stimulation has proven to trigger a more extensive visual cortical activation than in patients not using it (Plank et al., 2013); in particular, stimulation of the PRL was able in some studies to activate that very cortical area normally receiving input from the lesioned macula (Baker et al., 2008; Masuda et al., 2008; Schumacher et al., 2008; Plank et al., 2017) but not in all of them (Liu et al., 2010). However, in another one, PRL stimulation did not give any significant difference in response to the control group in whom a central scotoma was simulated.
– Even attention was found to influence cortical activation in the LPZ. In some JMD patients (Masuda et al., 2008; Liu et al., 2010), this area was activated during visual tasks, in contrast with passive viewing stimulation. It is not certain whether this finding can simply be justified by the unmasking of preexisting feedback signals from higher visual areas and by the upregulation of existing synaptic connections or instead if it can be a signal of a large-scale reorganization that occurred. It should be noted that there is one study that did not detect the activation of LPZ by the PRL by using only passive visual stimuli, without visual task.
– The nature of the stimulus seems also to influence brain response (Plank et al., 2017): object pictures were found to elicit stronger responses of the central representation area in the visual cortex than did the flickering checkerboard, probably due to feedback projections from higher visual areas.
– Finally, against the hypothesis of remodeling, it was pointed out by Haak et al. (2016) that a retinotopic organization of the visual cortex has proven to persist although long after the deafferentation generated by AMD, even if decreased in comparison to healthy controls.

### Possible Causes of Cerebral Changes in MD Patients

Causes of cerebral changes in MD patients appear therefore to be multifarious and not completely understood. Retinal degeneration in MD subjects led to a reduction of neuronal volume along specific parts of the visual pathway: as already mentioned above, functional deprivation is presumably responsible for a part of the structural changes, as also suggested by animal experimental models (Vercelli and Cracco, 1994; Palanza et al., 2002), maybe by anterograde transsynaptic degeneration (Medeiros and Curcio, 2001; Kim et al., 2002). However, it is not clear yet whether this could be the only explanation; other reasons might be its weight and whether the CNS changes in volume and density could also be associated with a plastic remodeling, for example, as a consequence of oculomotor training. In the analysis of functional studies, it has been reported that there is a variation not only in occipital cortical activation, as could be expected, but interestingly also in other areas not immediately related with the visual elaboration, suggesting that, if not a remodeling, at least an adaptation to the new situation with the involvement of other cortical functions had occurred in order to compensate for the pathological condition, for example, to support a more complex activity of fixation. Oculomotor training has proven to be able both to improve the patient’s performance and to cause functional and structural changes in the brain and cerebellum.

Extremely interesting was the analysis of cortical activity in relation to PRL: in patients able to use a PRL, its stimulation increased the occipital cortical activity and even caused the activation of LPZ, the cortical area normally activated by stimulation of the fovea being now damaged. This result strongly suggests the idea of a successful remodeling occurring in terms of receptive fields, but as rightly pointed out by some authors (Masuda et al., 2008), functional analysis does not allow us to discern whether this is related to a true neuronal plasticity or simply to preexisting conditions (feedback from extrastriate cortex, unmasking of preexisting task-dependent cortical signals that ordinarily are suppressed by the retinal visual input, and lateral connections). Even the results (Masuda et al., 2008) regarding how attention (visual task) affects the activation of visual cortex and LPZ do not clearly clarify whether such observations are related to a remodeling or an unmasking of preexisting synaptic connections.

It is, however, very important to emphasize as some results go in an opposite sense: Liu et al. (2010) and Baseler did not find any evidence of an increased activation of the visual cortex part normally receiving input from the lesioned retina, and Haak et al. (2016) showed that functional connectivity between V1 and V2–V3 was still largely retinotopically organized despite a prolonged lack of visual input due to MD. Is it therefore really possible that in an adult SNC a plastic remodeling can take place? Even though cerebral plasticity during childhood is a well-established phenomenon, adult plasticity remains actually elusive. The ability of the cerebral cortex to adapt to changes in visual experience and the mechanisms underlying this process are still highly debated (Wandell and Smirnakis, 2009). Nevertheless, many human studies have suggested that, during the life span, the visual cortex maintains the ability to structurally and functionally reorganize itself; two types of neuroplasticity are involved, although their borders are not well defined: structural and synaptic/functional plasticity. Structural plasticity refers to changes in axons, dendrites, and dendritic spines; synaptic plasticity refers to changes in synaptic activity (Rosa et al., 2013). Visual cortical plasticity was also proven in healthy subjects in the absence of visual loss as consequence of training (Berry and Nedivi, 2016).

We are therefore justified in assuming that cerebral remodeling, based on neuronal plasticity, could probably also take place in conditions of MD.

### Limits

It has been impossible to standardize the results available in literature due to the low sample size of the existing studies and the variability of intersubject anatomy, demographic characteristics, cortical reconstruction, and ROI identification. The extension and severity of maculopathy, the duration of the disease, and the type of lesions were also heterogeneous.

Moreover, as regards the fMRI studies, there was also a great variability in the kind of stimuli used, and the use of the task-related fMRI is another potential source of variability, if compared for example to the resting-state fMRI.

### Therapeutic Perspectives

Neuronal reorganization is undoubtedly useful in compensating for the deficit caused by MD and above all in being able to take full advantage of the rehabilitation possibilities, such as through oculomotor training aimed at using PRL, as described above. But would remodeling always be considered a positive occurrence? It is interesting to observe how the stability of visual neuronal organization may be beneficial: many of the most promising treatments aimed at restoring vision at a retinal level, such as antiangiogenic injections or more pioneering ones such as retinal prostheses, stem cell therapy, or even genetic therapy, rely on the assumption that cortical circuitry remains largely unchanged.

So such changes would first need to be reversed before the restored inputs could be normally processed. Structural changes also, especially in terms of volume reduction caused by the ceased trophic effect, could be a tough challenge, and we do not know at this time if it could be possible to bypass this obstacle or whether the visual brain would no longer be able to process retinal inputs appropriately.

That said, worthy of research is the possibility of stimulating neuronal plasticity to our advantage: perineuronal nets were found to control and contrast plasticity in the adult CNS (Sorg et al., 2016), as demonstrated by studies about neuronal axon regeneration. An eligible candidate to fight this effect seems to come from digestion of their glycosaminoglycan chains, thanks to the enzyme chondroitinase ABC (*ChABC*): interestingly, administration of ChABC after spinal cord injury permits some axon regeneration and greatly increases plasticity, and when combined with appropriate rehabilitation, ChABC treatment can lead to a considerable restoration of function, even in patients with neurodegenerative diseases (Fawcett, 2015). Since rehabilitation attempts through exercises and oculomotor training (Rosengarth et al., 2013; Plank et al., 2014, 2017) can be a worthwhile option but can achieve results only if these structural and functional modifications in the visual system occur, we can imagine, as a possible future therapeutic perspective, a combination of specific oculomotor and visual training and pharmacological stimulation of neuroplasticity aimed at obtaining a percentage of improvement in both AMD and JMD patients (Kwok et al., 2008; Fawcett, 2009; Yang et al., 2015; de Winter et al., 2016; Restani and Caleo, 2016; Foscarin et al., 2017).

## Conclusion

Through this systematic review, we highlighted how several brain modifications, both structural and functional ones, have been recorded in both AMD and JMD. Results were, however, heterogeneous and not always with unequivocal interpretations, not clearly demonstrating the existence of neuronal remodeling following macular damage, although it would seem that there exist more data in favor of this hypothesis than against it. It is quite possible that, after a retinal damage, both plastic remodeling and reorganization based on the unmasking of preexisting circuits may occur in these patients.

In subjects who have carried out ocular training exercises, for example, aimed at using a PRL as the new main area of light elaboration or obtaining stable eccentric fixation, the greatest cortical activities were recorded and achieved best performances in visual tasks. Although the possibility of a brain circuit remodeling would not be beneficial for all possible future therapeutic strategies, as it could hinder the correct processing of light signal once retinal function would be restored, it would support this kind of rehabilitation therapy which, as demonstrated, requires structural and functional modifications, as well as the involvement of upper cortical areas.

Further studies are therefore needed, preferably with greater sample size and more standardized in patient characteristics, disease entity, stimulus type, and analysis, in order to obtain more evidence of remodeling occurrence and how to use it in a therapeutic key, but we can imagine, as a possible future, a combination of specific oculomotor training and biochemical stimulation of neuroplasticity aimed at obtaining a percentage of improvement in both AMD and JMD patients.

## Author Contributions

All authors gave their substantial contribution to conception and design of the manuscript and to the acquisition and interpretation of data and materials. All authors gave their contribution in drafting the manuscript and in its critical revision for important intellectual content. All authors have approved the manuscript in its present form for publication. All authors agreed to be accountable for all aspects of the work in ensuring that questions related to the accuracy or integrity of any part of the work are appropriately investigated and resolved.

## Conflict of Interest

The authors declare that the research was conducted in the absence of any commercial or financial relationships that could be construed as a potential conflict of interest.
